# Generation of Stable cisPt Resistant Lung Adenocarcinoma Cells

**DOI:** 10.3390/ph13060109

**Published:** 2020-05-29

**Authors:** Nico Ruprecht, Lukas Hofmann, Martin Nils Hungerbühler, Christoph Kempf, Johannes Thomas Heverhagen, Hendrik von Tengg-Kobligk

**Affiliations:** 1 Department of Diagnostic, Interventional and Pediatric Radiology, Bern University Hospital, University of Bern, 3010 Bern, Switzerland; martin.hungerbuehler@dbmr.unibe.ch (M.N.H.); christoph.kempf@dbmr.unibe.ch (C.K.); Johannes.heverhagen@insel.ch (J.T.H.); Hendrik.vontengg@dbmr.unibe.ch (H.v.T.-K.); 2 Department of BioMedical Research, University of Bern, 3008 Bern, Switzerland; 3 Department of Pharmacology, School of Medicine, Case Western Reserve University, Cleveland, OH 44106, USA; lxh278@case.edu

**Keywords:** cisplatin, NSCLC, lung cancer, VRAC, resistance

## Abstract

Platinum compounds represent the backbone of combined chemotherapy protocols for advanced lung cancer. The mechanisms responsible for its frequent primary or acquired resistance to cisplatin (cisPt)-based chemotherapy remains enigmatic. The availability of two cell lines of the same origin, one resistant and the other sensitive, will facilitate research to reveal the mechanism of resistance formation. Lung adenocarcinoma cells, A240286S (A24), were cultivated in increasing cisPt concentrations over a prolonged time. After a significant increase in IC_50_ was measured, cultivation of the cells was continued in absence of cisPt and IC_50_s determined over a long period (>7 months). As a result, a cell line with lasting, high-level cisPt resistance, designated (D-)A24cisPt8.0, was obtained. The cells were cross-resistant to oxaliplatin and to pemetrexed at a low level. Previous publications have claimed that Leucine-rich repeat-containing protein 8 (LRRC8A and LRRC8D) of the volume-regulated anion channels (VRACs) affect cellular resistance to cisPt. Even though cisPt decreased LRRC8D expression levels, we showed by knockdown and overexpression experiments with LRRC8A and D that these proteins do not govern the observed cisPt resistance. The tumor cell sublines described here provide a powerful model to study the mechanisms of resistance to cisPt in lung cancer cells and beyond.

## 1. Introduction

With nearly 1.5 million new cases diagnosed each year worldwide, lung cancer is the most frequently diagnosed cancer in men and the leading cause of cancer death [1,2]. There are two main types of this disease, non-small cell lung cancer (NSCLC) and small cell lung cancer (SCLC). Eighty-five percent of all cases and thus the most abundant form of lung cancer is NSCLC [3]. Nearly 70% of patients diagnosed with NSCLC are found in the advanced or metastatic disease stage [4]. In spite of newly developed anti-cancer agents, the effect of platinum-based drugs to patients cannot be neglected. The use of Bevacizumab, a humanized monoclonal antibody that targets vascular endothelial growth factor (VEGF) improved responses and survival of patients only in combination with carboplatin and paclitaxel significantly [5]. As a consequence, platinum-based chemotherapy governs the first-line treatment [1,6].

The group around Barnett Rosenberg discovered the robust anti-proliferative effects of cisPt in the late 1960s. Formation of a complex between Pt(II) and nitrogen atoms of the nucleotide bases (platinum-DNA adducts) causes kinks and partial unwinding of the DNA helix [7]. This conformational change of the DNA halts the cell cycle and initiates programmed cell death [5,8]. Passive diffusion was assumed to be the only mechanism for cisPt entering the cell [9,10]. In recent years, however, alternative pathways such as the Copper transporter 1 (CTR1) were identified as an important transmembrane protein involved in cisPt uptake [11]. Furthermore, copper-extruding P-type ATPases were found to alter the export of cisPt too [12]. Recently, Planells-Cases et al. [13] described that the loss of LRRC8A and LRRC8D subunits of the volume-regulated anion channel (VRAC) affects cisPt uptake.

Inherent or acquired platinum resistance is a major limitation to improve long-term outcomes in cancer therapy. Recent discoveries were describing multiple novel resistance mechanisms [14,15,16,17]. These mechanisms with an effect on the development of cisPt resistance with clinical implications include decreased drug import, increased drug export, increased drug inactivation by detoxification enzymes, increased DNA damage repair, and inactivated cell death signaling, just to name the major possible mechanisms. To overcome cisPt resistance in cancer patients, numerous approaches have been undertaken [17]. Unfortunately, none of these approaches has been clinically implemented so far [1]. The recently proposed role of VRACs as entry for cisPt and carboplatin may appear as a promising new target to circumvent platinum-drug resistance in cancer patients [18,19].

The definition of sensitivity to cisPt used in the present report is based on the first performed identification of drug concentrations in vitro that correspond to clinical sensitivity of lung tumors. Ex vivo determined IC_50_ values for cisPt in endoscopic lung tumor specimens were correlated with effects of platinum-based combination chemotherapy on the respective primary tumors in vivo. Pretherapeutic ex vivo IC_50_ values for cisPt below 5 µM correlated with partial remission. At higher pretherapeutic IC_50_ values, the influence of the co-medication increased. Pemetrexed dominated in NSCLC, etoposide in SCLC [20]. Thus, lung tumors with IC_50_ values for cisPt below 5 µM are considered as sensitive to this drug.

The present report exclusively concerns the in vitro generated resistance to cisPt. Thereby, the platinum-sensitive lung adenocarcinoma wild-type (wt) cells from a suprarenal metastasis were exposed to cisPt. Most specimens of NSCLC are adenocarcinomas, but the presented findings are unrelated to therapeutically naïve tumors and restricted to cisPt resistance resulting from platinum-based chemotherapy. Sublines with decreasing cisPt responsiveness arose by stepwise increasing drug concentration. These sublines will facilitate analysis of the development as well as understanding the nature of mechanism(s) responsible for the acquisition of platinum resistance. Next generation sequencing analysis could reveal the specific and molecular details of gene expression patterns responsible for cisplatin resistance formation. In early stages, de-induction in the absence of drug revealed only temporary effects [21]. Clinically relevant degrees of resistance obtained at inducer concentrations above 4 µM persisted for almost one year and in its absence. This allows the identification of both therapeutic agents, whether or not they are cross-resistant to cisPt, and mechanisms of resistance to platinum derivatives that act in NSCLC.

## 2. Results

### 2.1. Immunocytochemical Verification of Lung Adenocarcinoma Properties of A24 wt Cells

**Figure 1:**
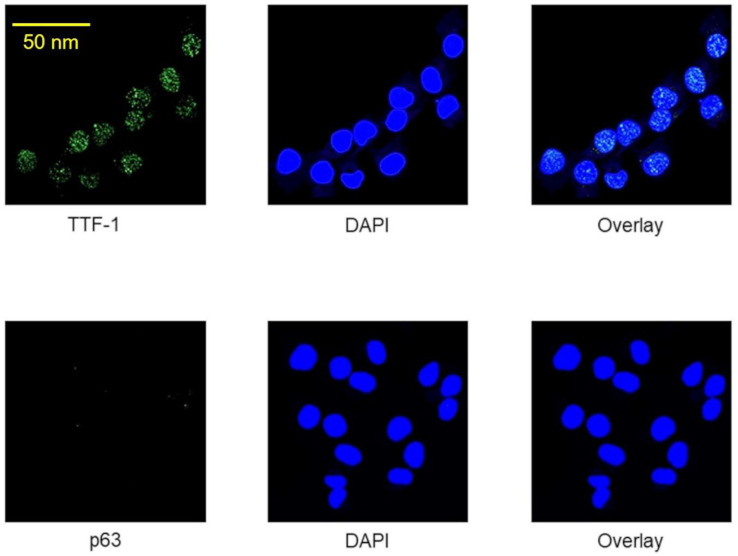
Immunostaining patterns of thyroid transcription factor 1 (TTF-1) and protein p63 in A24 wt cells.

In the present study, the lung adenocarcinoma origin of A24 wt cells was verified by immunofluorescence staining. Thyroid transcription factor 1 (TTF-1) was used as a specific marker for adenocarcinomas, while tumor protein p63 (p63) is a specific marker for squamous cell carcinomas [22]. A24 wt cells were fixed and stained with TTF-1 or p63. These staining demonstrated that A24 wt cells were positive for TTF-1 but negative for p63 (Figure 1).

### 2.2. Development, Levels, and Stability of Graded cisPt Resistance in A24cisPt and (D-)A24cisPt Sublines

**Figure 2:**
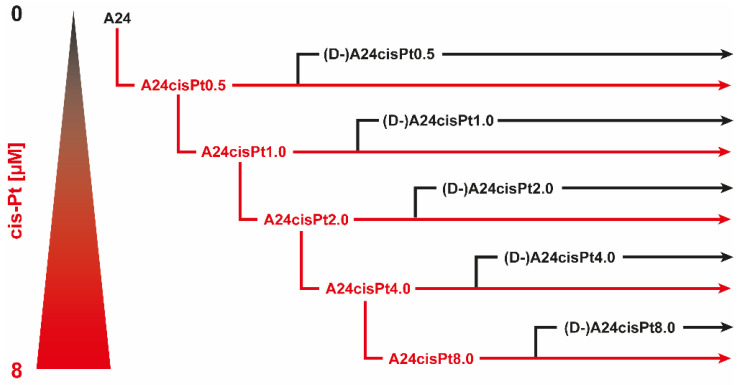
Schematic representation of A24cisPt and (D-)A24cisPt subline generation.

A24cisPt sublines with reduced cisPt sensitivity (A24cisPt2.0, A24cisPt4.0, and A24cisPt8.0) were sequentially derived from the wt A24 cell strain and exposed to their defining cisPt concentration for several months, followed by a de-induction phase without cisPt exposure as described in materials and methods (Figure 2).

**Figure 3:**
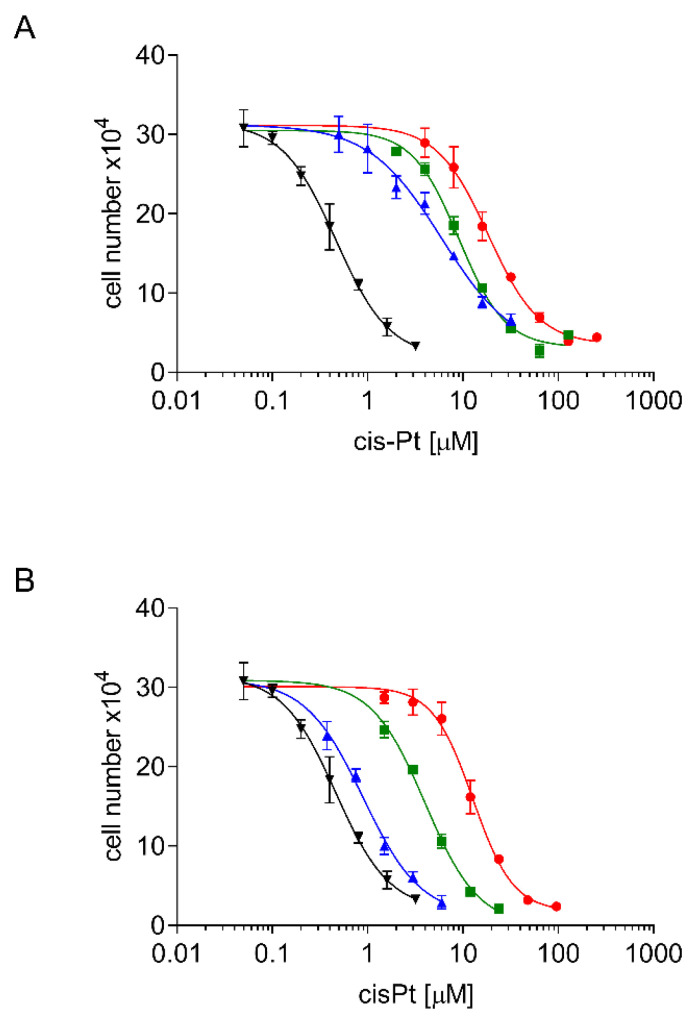
Dose response curves of induced (**A**) and de-induced (**B**) A24 sublines. Cells were seeded at densities of 2–8 × 10^4^ mL^−1^ and subsequently grown in cisplatin containing medium (0–512 µM). Cell densities were measured after three days. A24 (-**▼**-), A24/(D-)A24cisPt2.0 (-**▲**-), A24/(D-)A24cisPt4.0 (-**■**-), A24/(D-)A24cisPt8.0 (-**●**-). Data are presented as mean ± standard deviation (SD). *n* > 10.

Dose-response curves of cisPt for sublines the A24 wt cell strain, for its sublines A24cisPt2.0, A24cisPt4.0, A24cisPt8.0, and for their de-induced (D-) counterparts were derived from at least three individual experiments and are shown in Figure 3.

Table 1 shows a comparison between IC_50_ values for cisPt, population doubling time of the A24 wt adenocarcinoma cell strain subline (column 1), and IC_50_ values for cisPt of the A24cisPt, (D-)A24cisPt sublines, and the population times observed in the latter.

There were 13-fold, 20-fold, and 40-fold increases in the IC_50_ values of the A24cisPt2.0, A24cisPt4.0, and A24cisPt8.0 sublines, compared to the A24 wt subline. But de-induced (D-)A24cisPt2.0, (D-)A24cisPt4.0, and (D-)A24cisPt8.0 sublines still showed two-fold, nine-fold, and 30-fold increases in IC_50_ values, compared to the A24 wt subline. After almost one year in the absence of cisPt, the calculated IC_50_ value was still 12.74 ± 0.78 μM for the (D-)A24cisPt8.0 subline.

### 2.3. Cross Resistance of A24cisPt and (D-)A24cisPt Sublines to Oxaliplatin and Pemetrexed

**Figure 4:**
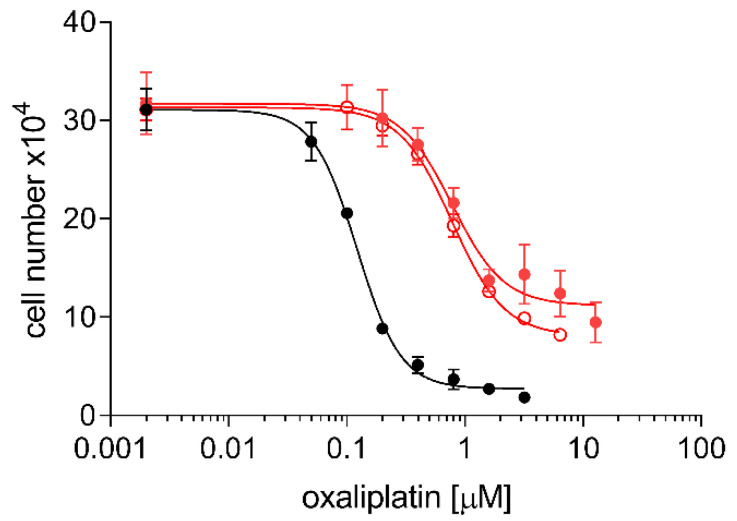
Cross resistance of A24cisPt8.0 and (D-)A24cisPt8.0 cells toward oxaliplatin. Cells were seeded at densities of 2–8 × 10^4^ mL^−1^ and subsequently grown in medium containing oxaliplatin (0–12.8 µM). Cell densities were measured after three days. A24 (-●-), A24cisPt8.0 (-●-), (D-)A24cisPt8.0 (-**○**-). Data are presented as mean ± SD. *n* > 10.

Figure 4 shows cross-resistance to oxaliplatin of the A24cisPt8.0 and (D-)A24cisPt8.0 sublines. Corresponding IC_50_ values for oxaliplatin were 0.12 ± 0.005 μM for wt A24, 0.78 ± 0.11 μM for A24cisPt8.0 and 0.78 ± 0.05 μM for (D-)A24cisPt8.0. The IC_50_ values were significantly different form wt A24, *p* < 0.0001 (A24cisPt8.0), and *p* < 0.0001 ((D-)A24cisPt8.0).

**Figure 5:**
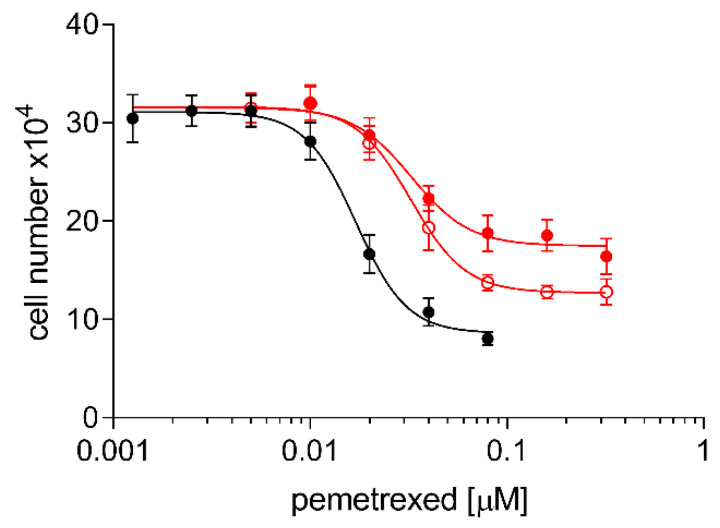
Cross resistance of A24cisPt8.0 and (D-)A24cisPt8.0 cells towards pemetrexed. Cells were seeded at densities of 2–8 × 10^4^ mL^−1^ and subsequently grown in medium containing pemetrexed (0–0.32 µM). Cell densities were measured after three days. (-●-), A24cisPt8.0 (-●-), (D-)A24cisPt8.0 (-**○**-). Data are presented as mean ± SD. *n* > 10.

Figure 5 shows significant cross-resistance of both the A24cisPt8.0 and the (D-)A24cisPt8.0 sublines to pemetrexed but at a very low level. IC_50_ values of pemetrexed were 0.017 ± 0.001 μM for wt A24, 0.033 ± 0.002 μM for A24cisPt8.0 and 0.033 ± 0.002 μM for (D-)A24cisPt8.0. The IC_50_ values were significantly different from wt A24, *p* < 0.0001 for A24cisPt8.0 and (D-)A24cisPt8.0.

### 2.4. Expression of VRAC Subunits in A24 wt, A24cisPt, and (D-)A24cisPt Cells

In an initial attempt to shed light on the mechanism of resistance formation, we focused on the LRRC8 proteins of VRACs. LRRC8 transporter proteins of VRACs were claimed by Planells-Cases et al. [13] to have a significant clinical impact in cellular uptake of platinum, to influence the efficacy of platinum-based drugs, and to require adjustments of the treatment strategy.

**Figure 6:**
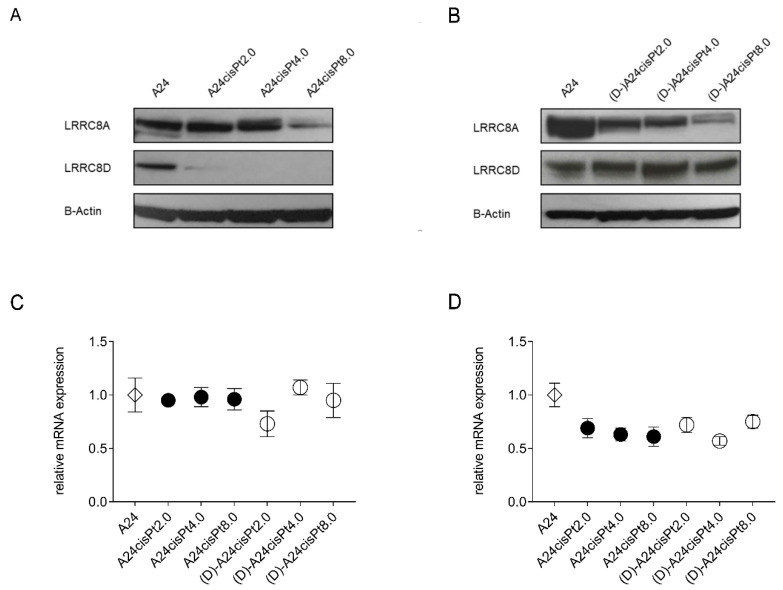
Western Blot illustrating LRRC8A and LRRC8D subunits expression in induced (**A**) and de-induced (**B**) A24 sublines. mRNA levels of LRRC8A (**C**) and LRRC8D (**D**) were quantified by RT-qPCR. Western blots are representative of 3 independent repeated experiments. Data are presented as mean ± SD. *n* = 3.

Western blot analysis of VRAC subunits LRRC8A and LRRC8D expression levels in A24 wt cells are shown in Figure 6. Expression levels of both subunits were affected differently in A24cisPt sublines according to cisPt concentrations. The LRRC8D expression strongly decreased with increasing cisPt levels. In the A24cisPt2.0 subline, LRRC8D was barely detectable and was abolished in the A24cisPt4.0 and A24cisPt8.0 sublines. Interestingly, LRRC8D expression levels returned to wt level in all de-induced (D-)A24cisPt sublines. In comparison, expression levels of the LRRC8A subunit was solely changed in A24cisPt8.0 and the de-induced (D-)A24cisPt8.0. A partial decrease of LRRC8A suppression was observed and remains unchanged with de-inducing conditions.

Normalized gene expression of LRRC8A and LRRC8D in resistant cisPt were compared to the cisPt sensitive A24 wt subline by RT-qPCR. The mRNA levels of LRRC8A in induced or in de-induced sublines were almost identical (96% or 92%, respectively) compared to the A24 wt (Figure 6C). However, the average mRNA level of LRRC8D in induced or de-induced sublines dropped significantly to 64% or 68%, respectively (Dunnett’s test, *p* Value = 0.0016 or 0.0028, respectively) (Figure 6D).

### 2.5. CisPt Response of siLRRC8A or siLRRC8D Transfected A24 wt Cells

**Figure 7:**
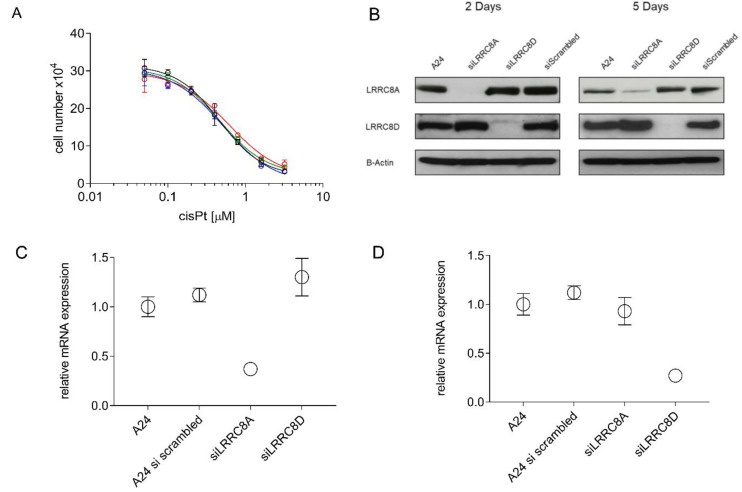
W Suppression of LRRC8A or LRRC8D subunits in A24 wt cells by siRNA. A24 wt cells were cultivated for two days in absence or presence of siRNA, respectively, and subsequently seeded at densities of 2 × 10^4^ mL^−1^ and grown in cisPt containing medium (0–3.6 µM). Cell densities were measured after three days. wt (-**○**-), LRRC8A-siRNA (-**○**-), LRRC8D-siRNA (-**○**-), scrambled siRNA (-**○**-) (**A**). Knock down efficiency of the LRRC8 protein was checked by SDS-PAGE and western blotting (**B**). mRNA levels of LRRC8A (**C**) and LRRC8D (**D**) was quantified by RT-qPCR. Western blots are representative of three independent repeated experiments. Data are presented as mean ± SD. n equal 3 for mRNA quantification and > 10 for IC_50_.

IC_50_ values for cisPt were determined to test whether siRNA-mediated down regulation of LRRC8A or LRRC8D affects the phenotypical cisPt response in A24 wt cells. Calculated IC_50_ values were 0.47 ± 0.03 µM and 0.61 ± 0.01 µM for LRRC8A and LRRC8D knockdown cells, respectively. Thus, being almost identical to those observed in siRNA-untreated cells (Figure 7A). Suppression of LRRC8A or LRRC8D subunits of VRAC resulting of siRNA-mediated knockdown were confirmed by RT-qPCR and Western blotting (Figure 7B–D).

### 2.6. CisPt Response in Transfected (D-)A24cisPt8.0 Cells Overexpressing LRRC8A or LRRC8D

As reported previously, LRRC8A is required to transport LRRC8D to the plasma membrane [23]. Since LRRC8A is significantly reduced in (D-)A24cisPt8.0 cells (Figure 6B), overexpression experiments were performed using both wt LRRC8A and D-containing plasmids. The latter was used to exclude the possibility that the LRRC8D was mutated in the newly developed (D-)A24cisPt8.0 cells.

**Figure 8:**
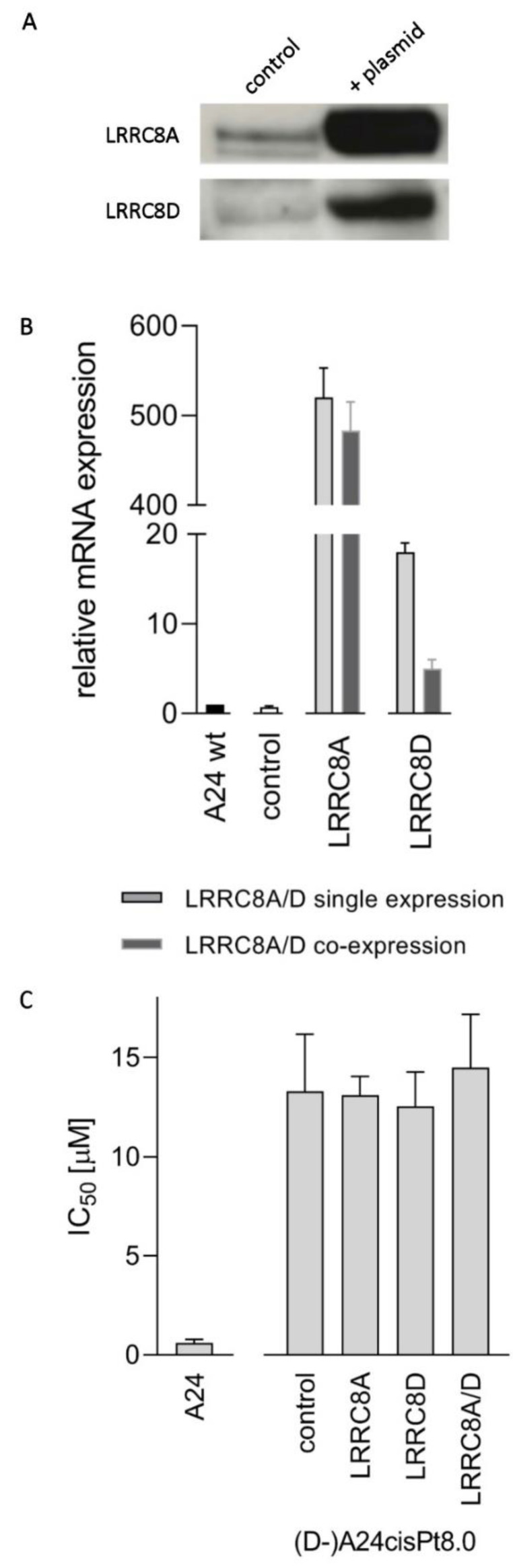
Overexpression of LRRC8A and LRRC8D in (D-)A24cisPt8.0 cells. (D-)A24cisPt8.0 cells were transfected with LRRC8A and / or LRRC8D cDNAs using Lipofectamine™ 2000. As a control, the cells were incubated with Lipofectamine only. Expression was tested by either Western Blot analysis (**A**) or RT-qPCR (**B**). Subsequent to expression, the IC_50_ values for cisPt (**C**) were determined as described in materials and methods. Western blots are representative of three independent repeated experiments. Data are presented as mean ± SD. n equal 3 for mRNA quantification and >10 for IC_50_.

Cells were transfected as described in material and methods to verify whether cisPt resistance is reversed in (D-)A24cisPt8.0 cells by overexpression of LRRC8A and/or LRRC8D. Successful overexpression of LRRC8A and/or LRRC8D subunits of VRAC was tested and confirmed by RT-qPCR and Western blotting. As depicted in Figure 8A LRRC8A appeared in transfected cells and was highly overexpressed compared to A24 wt cells as demonstrated with RT-qPCR (Figure 8B). LRRC8D is present in equivalent amounts in A24 wt and (D-)A24cisPt8.0 cells as judged by Western blot (Figure 6B). LRRC8D was overexpressed successfully, verified by RT-qPCR. Determination of IC_50_ values showed no difference in LRRC8A and/or LRRC8D overexpressing cells compared to the (D-)A24cisPt8.0 wt cells (Figure 8C). Corresponding IC_50_ values were 13.1 ± 1.0 µM in (D-)A24cisPt8.0 wt cells and 12.6 ± 1.7 µM and 13.3 ± 2.9 µM in LRRC8A or LRRC8D overexpressing cells, respectively. An IC_50_ value of 14.5 ± 2.7 µM was measured when LRRC8A and LRRC8D were co-expressed.

## 3. Discussion

It is a fundamental requirement to fully understand the underlying mechanism of cisPt resistance in NSCLC. Only this understanding will allow us to successfully deploy cisPt as the treatment of choice for patients in first-line combination regiments of NSCLC in the future [1]. 

A24 wt cells were established from a hematogenous suprarenal metastasis of a lung adenocarcinoma. It is well known that tumors are heterogeneous and, in consequence, cell lines derived thereof are heterogeneous, too [24]. A24 resistant sublines were obtained by cultivating in presence of cisPt over a prolonged time allowing divergent evolution for the acquisition of a cisPt resistance. Resistance could have been acquired by either molecular changes within the entire cell population over time or alternatively by selection over time of preexisting resistant cells. The latter would certainly lead to a reduction in heterogeneity.

The present study is chiefly concerned with establishing the framework required to analyze, in NSCLC, the development of resistance to cisPt during platinum-based combination chemotherapy. Hence, sensitivity as well as resistance to cisPt were considered here as manifestations of the drug’s systemic toxicity. At the organism level, toxicity is tolerable in drug-sensitive settings and becomes intolerable in drug-resistant ones. The upper limit of cisPt tolerance in lung cancer patients was shown to correspond to a plasma level of 6.67 µM cisPt [25]. Different cisPt resistant cell lines have been already established and described [26,27,28]. However, to our knowledge, cell lines resistant to cisPt, which have been growing in absence of drug over a period of more than seven months, have not been previously published [24]. Strictly observing flavin-protecting procedures to prevent photochemical artifacts throughout the study should have been decisive for the survival of tumor cells at high cisPt concentrations [29,30]. The highly resistant A24cisPt8.0 subline is 40-fold more resistant to cisPt when compared to its parent line. Interestingly, the de-induced (D-)A24cisPt8.0 subline shows a 30% decrease of resistance as compared to the induced A24cisPt8.0 subline. This finding suggests that acquired cisPt resistance in A24 sublines is only partial lost in drug free medium. Similar findings were described in Adriamycin resistant cell lines, where 40 to 50% loss of resistance have been observed [31,32]. However, this effect is less pronounced in de-induced sublines of lower concentrations such as (D-)A24cisPt2.0 and (D-)A24cisPt4.0. We suggest that there is a threshold of cisPt concentration in induced A24 sublines which results in an acquired and sustained resistance in de-induced sublines.

In a systematic review of the literature performed by Stordal et al., a cross-resistance between cisPt and oxaliplatin was suggested. They described cross-resistance in low-level platinum resistant cell lines and in patients with cisPt resistant cancers [33]. In our study, induction of cisPt resistance in A24 cells displayed a minimal cross-resistance to oxaliplatin. A four-fold oxaliplatin resistance was determined in the A24cisPt8.0 subline and the (D-)A24cisPt8.0 subline when compared to their parent line. High levels of cisPt resistance in cisPt resistant A24 sublines were associated with marginal level of resistance to oxaliplatin. Similar findings were described by others [34,35,36]. Zhang et al. have demonstrated that pemetrexed-resistant NSCLC are 2.1 to 4.2-fold more resistant to cisPt when compared to its parent line [37]. In our study, a maximum of two-fold pemetrexed resistance was determined in the A24cisPt8.0 subline and the (D-)A24cisPt8.0 subline when compared to their parent cell line. These results support the conclusion that the acquired resistance mechanism in A24cisPt sublines also contributes to an oxaliplatin and pemetrexed resistance albeit to a lesser extent. However, it is highly speculative to assume that these sublines are multi-drug-resistant.

In a clinical study on advanced, chemotherapeutically naïve lung tumors, pre-therapeutic IC_50_ values for cisPt were determined in small bioptic tumor specimens, and biometrically compared to the radiologically determined effects of platinum-based combination chemotherapy on the respective primary tumors. Procedures used were as in the present study. All tumors with IC_50_ values of ≤5 µM for cisPt responded to platin-based combination chemotherapy with partial remission. Higher IC_50_ values of up to 7 µM corresponded to tumors responding with partial remission or with no change. The extremely variable therapeutic effects on tumors with pre-therapeutic IC_50_ values for cisPt above 7 µM resulted either from action of additional drugs used for the platinum-based combination chemotherapy (e.g., pemetrexed) or were due to interaction with the obligatory platinum drugs. Pre-therapeutic IC_50_ values above 20 µM for cisPt were frequently observed [20]. Accordingly, in the present study, lung adenocarcinoma cells with IC_50_ values for cisPt below 5 µM, between 5 µM and 7 µM, and above 7 µM were respectively considered as being sensitive, intermediately responsive, or definitively resistant to cisPt.

Numerous cellular mechanisms related to cisPt resistance are known [17,38]. Prevention of drug uptake by cancer cells is one strategy to become drug resistant. The copper transporter 1 (CTR1) has been successfully identified as transporter for the uptake of polar cisPt into the cytoplasm by several studies [39,40,41]. Conversely, a CTR1 dependent transport was disclaimed by Ivy and Kaplan. Their observations support a non-protein-mediated pathway [42]. Furthermore, Planells-Cases et al. have demonstrated that the loss of LRRC8A and LRRC8D subunits of the heteromeric volume-regulated anion channels (VRACs) in haploid KBM7 cells is related to resistance to cisPt. Similar results were obtained by Sorensen and collaborators [43,44,45]. It was suggested that loss of these subunits contribute to cisPt resistance due to decreased cellular uptake of the cytostatic drug. This is in agreement with our observation that in cisPt induced A24 sublines the LRRC8D subunit is downregulated on the protein level (Figure 6A). Downregulation of LRRC8A is less pronounced than LRRC8D (Figure 6B). Planells-Cases and coworkers [13] also observed, a correlation between LRRC8D downregulation and poor survival of Pt drug-treated patients, but not so with LRRC8A. Indeed, normalized gene expression of LRRC8D in induced and de-induced cisPt A24 sublines was downregulated by 33.6 ± 6%. Therefore, the expression profile of LRRC8D mRNA may predict the sensitivity of cancer cells to cisPt as described by Planell-Cases and coworkers [13]. In contrast, the gene expression of LRRC8A in induced and de-induced cisPt A24 sublines remained unaffected when compared to the parental A24 cell line. Taken together these findings so far may suggest that LRRC8 could be involved in cisPt resistance. However, the fact that in de-induced cells, that remained cisPt resistant protein expression of LRRC8D fully recovered (Figure 6B) suggested that resistance formation does not go along with the loss of this protein in the A24 sublines.

Therefore, knockdown experiments using siRNA against either LRRC8A or LRRC8D were carried out in wt A24 cells. Success of the knockdown was confirmed by RNA analysis by RT-qPCR and Western blot (Figure 7A–C). The use of scrambled siRNA showed no effect. Functional testing performed on these knockdown cells resulted in identical sensitivity of these cells towards cisPt compared to wt A24 cells. Additionally, no change in response to cisPt was observed when LRRC8A and/or LRRC8D, were overexpressed in (D-)A24Pt8.0 cells (Figure 8). Co-transfection was performed because LRRC8D remains intracellular if not co-transfected with LRRC8A [23,46]. Overexpression experiments of LRRC8D exclude that the developed resistance is due to a mutation affecting these proteins. These results clearly demonstrate that the putatively mentioned involvement of LRRC8 in resistance referred to above cannot be sustained in A24 sublines. In summary, it can be stated that loss of LRRC8A or LRRC8D does not govern the cisPt resistance in A24 cells. Hence, these findings are in contradiction to previously published studies for other cells [13,43,44,45]. Currently any explanation of this discrepancy would be highly speculative.

In conclusion, the here established and described A24cisPt cells might serve as a powerful model to study the molecular mechanism of the development of resistance to cisPt and potential re-sensitization mechanisms. In addition, future molecular characterization of these cell lines might also lead to discovery of biomarkers for cisPt resistance and/or resistance development in NSCLC.

Further, availability of stable resistant de-induced sublines allows for in vitro investigations of the cellular cross-resistance to cisPt of other cytostatic drugs. However, the limitation of the established cisPt resistant sublines is that they cannot reflect the microenvironment of a tumor.

In conclusion, we have established cisPt resistant NSCLC sublines. Molecular changes at the respective intermediate resistance levels of 0–8 µM can be identified by sequencing and analyses of the effects of knockdown and overexpression in resistant cells with increasing cisPt levels. The obtained resistance was stable over a period of almost one year and continues. The here established and described A24cisPt cells might serve as a powerful model to study the molecular mechanism of the development of resistance to this drug and potential re-sensitization mechanisms. Further, the availability of stable resistant de-induced sublines allows in vitro investigations of the cellular cross-resistance of other cytostatic drugs to cisPt.

## 4. Materials and Methods

### 4.1. Platinum Derivatives and Pemetrexed

Pharmaceutical preparations of cisPt (CISplatin Sandoz^®^, i.v. Infusion concentrate) and oxaliplatin (OXALIplatin Sandoz^®^, i.v. Infusion concentrate) were purchased from Galenica AG (Bern, Switzerland). Pharmaceutical preparation of pemetrexed (Alimata 500 mg) was obtained from Eli Lilly Nederland B.V. (Houten, Nederland). The aqueous solutions of cisPt were diluted with fresh medium to the desired concentrations directly before use. pemetrexed was dissolved in sodium chloride solution for injection to 25 mg/mL and subsequently diluted with fresh medium to the appropriate concentrations just before use.

### 4.2. Metastatic Lung Adenocarcinoma Cells and Cell Cultivation

The cell line A240286S (A24) used was provided by Dr. C. Granzow and has been described previously [30,47]. A24 wt cell strain was mistakenly assumed to origin from the lung metastasis of a hypernephroma [47]. Subsequent reevaluation of the clinical records, however, attributed the A24 cells to originate form a presumably hematogenous suprarenal metastasis of a lung adenocarcinoma, though, without molecular proof. [30].

The A24 subline and any further sublines used in the course of the present study were cultured in RPMI 1640 without riboflavin, phenol red and antibiotics, buffered with 4.5 mM HEPES (BioConcept, Allschwil, Switzerland), supplemented with 10% (v/v) fetal bovine serum (FBS) (Thermo Scientific, Waltham, MA, USA) as the only source of flavins and 13.5 mM NaHCO_3_. At subcultivation, cell monolayers were rinsed with PBS and exposed for 3 min to StemPro Accutase Cell Dissociation Reagent (Thermo Scientific, Waltham, MA, USA) at 36.5 °C. Detached cells were re-suspended in culture medium. Cell densities were determined using Moxi flow cytometer according to manufacturer instructions (Orflo Technologies, Ketchum, ID, USA). Experiments and subcultivations were performed using light with wavelengths above 520 nm to prevent photochemical artifacts [29]. Culture vessels and test plates were incubated in the dark at 36.5 °C below 3.5% CO_2_ (vol/vol) and humidified air. Absence of mycoplasma was checked regularly.

### 4.3. Immunofluorescence

A24 wt cells were washed with PBS and fixed with 4% paraformaldehyde. Fixed cells were incubated with monoclonal anti-TTF-1 antibody or anti- p63 antibody (1:500 dilution, Abcam, Cambridge, UK) for 1 h at room temperature. After incubation, the cells were washed and stained using Alexa™ 488-conjugated goat anti-rabbit secondary antibody (1:500 dilution, Molecular Probes, Eugene, OR, USA) for 1 h at room temperature in the dark. Then, the cells were washed, followed by incubation with DAPI for 1 min and mounted with Dako fluorescent mounting medium (Dako, Carpinteria, CA, USA). Fluorescence images were obtained by using confocal laser scanning microscopy with a 63× magnification objective (Zeiss LSM 880 confocal microscope with Airyscan).

### 4.4. Induction, Maintenance, and De-Induction of cisPt Resistance

Covering the range of 0.5 µM, 1.0 µM, 2.0 µM, 4.0 µM, and 8.0 µM, five A24cisPt sublines with reduced cisPt sensitivity (A24cisPt0.5, A24cisPt1.0, A24cisPt2.0, A24cisPt4.0, and A24cisPt8.0) were sequentially derived from the wt A24 cell strain. The corresponding cisPt concentration was added to the culture medium during the stepwise duplication at branching-off (Figure 2). After branching-off, A24cisPt sublines were propagated with the cisPt concentration indicated until the temporarily reduced growth rates levelled, thereby indicating successful subline establishment and enabling the next branching-off. Newly established sublines were then exposed to their defining cisPt concentration for several months, followed by a de-induction phase without cisPt exposure (Figure 2). In the present study, A24cisPt2.0, A24cisPt4.0, and A24cisPt8.0 sublines and the corresponding de-induced (D-)A24cisPt2.0, (D-)A24cisPt4.0, and (D-)A24cisPt8.0 sublines were included, along with the wt A24 subline.

### 4.5. IC_50_ Determination

Cytostatic drug response was quantified as described by Heuser et al. [30] with minor modifications. Wt A24 single cell suspensions were freshly prepared using Accutase as described above, and diluted in culture medium to a cell density of 2 × 10^4^ cells/mL. 150 µl of this suspension was combined with 150 µl of twice the cisPt, oxaliplatin or pemetrexed concentration in a 96-well microtiter plate (TPP, Trasadingen, Switzerland). For the sublines A24cisPt2.0, A24cisPt4.0, A24cisPt8.0, and the de-induced (D-) counterparts, cell densities of 4 × 10^4^, 8 × 10^4^, and 8 × 10^4^ cells/mL were used. Test plates were incubated with standard conditions for 72 h. Cell densities were determined in a Casy I cell analyzer using Casystat software (OLS OMNI Life Science, Bremen, Germany). Drug concentrations leading to 50% growth inhibition compared to controls were calculated using GraphPad Software (La Jolla, CA, USA). Each experiment was done in triplicates.

### 4.6. Western Blotting

Protein expression was analyzed by SDS-Page and Western blotting. Briefly, after washing the cells in ice-cold PBS, the cell pellet was re-suspended in Triton based RIPA lysis buffer supplemented with the protease inhibitor cocktail, cOmplete Mini, EDTA-free (Merck KGaA, Darmstadt, Germany). Lysates were centrifuged at 8′609 rcf and the supernatant was mixed with NuPAGE™ LDS Sample Buffer supplemented with NuPAGE™ Sample Reducing Agent (Thermo Scientific, Waltham, MA, USA). SDS-PAGE was performed using NuPAGE™ 4–12% Bis-Tris gels and NuPAGE™ MOPS SDS running buffer (Thermo Scientific, Weltham, MA, USA). Proteins were transferred using activated porous 0.45 μm polyvinylidene fluoride (PVDF) membranes (Thermo Scientific, Weltham, MA, USA). After blocking the membranes in TBS-T (0,15 M NaCl, 50 mM Tris-HCl, 0.1% Tween 20, pH 7.6) containing 5% milk powder and 1% bovine serum albumin (Merck KGaA, Darmstadt, Germany), a mouse MAb against LRRC8A (8H9) and a mouse MAb against LRRC8D (A-12) (Santa Cruz Biotechnology, Dallas, TX, USA) were used to detect subunit composition of VRAC. Rabbit PAb to β-Actin (Abcam, Cambridge, UK) was used as loading control. After washing the membranes with TBS-T, they were incubated with secondary horse radish-peroxidase conjugated anti-mouse or anti-rabbit antibodies (Agilent technologies Dako, Santa Clara, CA, USA). Membranes were developed using SuperSignal^®^ West Dura Extended Duration Substrate (Thermo Scientific, Waltham, MA, USA) and visualized by exposing the membranes to Hyperfilm ECL (GE Healthcare, Buckinghamshire, UK).

### 4.7. siRNA Cell Assay in A24 wt Cells

siLRRC8A (5′-AAAGUCAUCGACCGUCAGUTT-3′) or siLRRC8D (5′-CCAUGCAACUUACCAAAGATT-3′) were diluted in RPMI 1640 supplemented with Lipofectamin^®^ RNAiMAX (Thermo Scientific, Weltham, MA, USA) to a final concentration of 27 nM in a six-well plate. As negative control, cells were transfected with scrambled siRNA (5′-GCCACACGAUAACCUAACUTT-3′). Small interfering ribonucleic acids were purchased from Microsynth, Balgach, Switzerland. Cells were re-suspended in RPMI 1640 containing 10% FBS. This cell suspension was added to the well and incubated with standard conditions for 48 h. Transfected cells were used for IC_50_ determinations for cisPt as described above.

### 4.8. Overexpression Cell Assay in (D-)A24cisPt8.0 Cells

One day before transfection, 8 × 10^5^ cells were plated in a 6- well plate with RPMI 1640 containing 10% FBS. Cells were transfected according to manufacturer’s standard protocols using Lipofectamine™ 2000 Transfection Reagent (Thermo Scientific, Waltham, MA, USA) with 4.0 µg Human LRRC8A or LRRC8D cDNAs cloned into pCMV6 (OriGene Technologies, Rockville, MD, USA). 2.0 µg of Human LRRC8A and LRRC8D cDNA were used to co-express LRRC8A and LRRC8D. As a control, (D-)A24cisPt8.0 cells were incubated with Lipofectamine only. IC_50_ determinations of transfected cells were carried out as described above.

### 4.9. Reverse Transcription Real-Time Quantitative Polymerase Chain Reaction (RT-qPCR)

Total poly-A-mRNA was isolated from cells with a Dynabeads mRNA direct Kit- (Thermo Scientific, Waltham, MA, USA). The mRNA of LRRC8A, LRRC8D and β-actin was quantified with a one-step RT-qPCR using Luna^®^ Universal Probe One-Step RT-qPCR Kit (Ipswich, MA, USA) on a BioRad CFX real time system (BioRad, Hercules, CA, USA). Primers were designed to amplify a 128 nt-long LRRC8A fragment, a 122 nt-long LRRC8D cDNA fragment and a 127 β-Actin fragment:

LRRC8A forward (5′-GGTTTGCCAAGTACTTCCCCTAC-3′),
LRRC8A reverse (5′-CTTCAGCAGGATAGACACAAAGTG-3′),
LRRC8D forward (5′-CATGCAACTTACCAAAGATCAGGTG-3′),
LRRC8D reverse (5′-CTGCTTCCATCTTTGGGATGTTG-3′),
β-Actin forward (5′-GATGGTGGGCATGGGTC-3′) and
β-Actin reverse (5′-GATTTTCTCCATGTCGTCCCAG -3′).

LRRC8A and LRRC8D mRNA expression were normalized to β-actin mRNA expression for each sample using the BioRad CFX Maestro software.

### 4.10. Statistics

Comparison of IC_50_ values (wt vs sublines) and mRNA expression values and statistical analysis were performed using Dunnett’s test and GraphPad Prism 8.0 Software [48].

### 4.11. Ethical Statements

All experiments were performed with cell cultures. The cell line A240286S (A24) used was provided by Dr. C. Granzow and has been described previously [30,47]. Since no patients were involved in these studies, no ethics committee approval is required.

## Figures and Tables

**Table 1:** Cell pharmacological parameters of the metastatic wt A24 lung adenocarcinoma cell strain and of its sublines with induced and with de-induced resistance to cisPt.

Starting Subline	A24 wt	A24 wt	A24cisPt0.5	A24cisPt1.0	A24cisPt2.0	A24cisPt4.0
cisPt concentration exposed to [µM]	n. a.	0.5	1.0	2.0	4.0	8.0
Branching-off after (months)	n. a.	2	2	3	4	12
Resulting subline	n. a.	A24cisPt0.5	A24cisPt1.0	A24cisPt2.0	A24cisPt4.0	A24cisPt8.0
IC_50_ cisPt at branching-off time	0.46 ± 0.05	2.13 ± 0.62	2.97 ± 0.81	6.03 ± 1.39	9.09 ± 0.56	18.49 ± 1.96
De-induction period (months)	n. a.	3	3	7	7	>7
De-induced subline	n. a.	(D-)A24cisPt0.5	(D-)A24cisPt1.0	(D-)A24cisPt2.0	(D-)A24cisPt4.0	(D-)A24cisPt8.0
IC_50_ cisPt [µM]	n. a.	0.89 ± 0.22	2.53 ± 0.84	0.99 ± 0.07	4.02 ± 0.23	12.74 ± 0.78
Population doubling time (h)	20.5	n. d.	n. d.	25	31	42

n. a.: not applicable; n. d.: not done; data are presented as mean ± SD. n equal 6 for doubling time and >10 for IC_50_.

## Abbreviations

cisPt	Cisplatin
wt	Wild-type
A24	A240286S
NSCLC	Non-small cell lung cancer
SCLC	Small cell lung cancer
TTF-1	Thyroid transcription factor 1
LRRC8A	Leucine-rich repeat-containing protein 8A
LRRC8D	Leucine-rich repeat-containing protein 8D
p63	Tumor protein p63
VEGF	Vascular endothelial growth factor
VRACs	Volume-regulated anion channels
